# Challenges and Progress in General Data Protection Regulation Implementation in Romanian Public Healthcare

**DOI:** 10.7759/cureus.78008

**Published:** 2025-01-26

**Authors:** Iulian V Nastasa, Florentina-Ligia Furtunescu, Dana G Mincă

**Affiliations:** 1 Department of Management and Public Health, Carol Davila University of Medicine and Pharmacy, Bucharest, ROU; 2 Department of Public Health, Carol Davila University of Medicine and Pharmacy, Bucharest, ROU

**Keywords:** gdpr, healthcare, privacy, privacy concerns in medical communication, public health

## Abstract

Abstract: The increasing integration of technology in European healthcare systems, including Romania, has highlighted the critical need for robust data privacy regulations under the General Data Protection Regulation (GDPR). This regulation is crucial for managing patient data and protecting individual rights. However, Romania faces challenges in implementing GDPR due to differing levels of awareness and the rapid advancement of healthcare technologies (e.g., lack of infrastructure or highly trained IT personnel).

Methods: The study uses a privacy-focused survey to assess healthcare professionals' compliance, awareness of GDPR principles, and challenges in implementing GDPR requirements. The survey was distributed to 108 Romanian healthcare professionals during an economics and healthcare management course organized between July and September 2024, ensuring anonymity and informed consent.

Results: While many practitioners demonstrate a foundational understanding of GDPR principles, gaps exist in practical applications, particularly in clinical settings. The digitalization of public institutions in Romania has been found to reduce the risk of confidentiality from a GDPR perspective, as evidenced by the responses of 82% of participants. Ethical concerns are cited as a significant rationale for not permitting exceptions. Primary challenges recognized by healthcare professionals in the implementation of GDPR requirements include accountability, communication, high volume of consents, laborious administrative duties, abusive use of GDPR, restricted access to specific health data, and administrative tasks. Healthcare professionals in Romania, compared to professionals in other European Union (EU) member states, face GDPR implementation challenges due to resource constraints and cultural barriers, but opportunities include increasing patient trust, innovation, international collaboration, and raising awareness.

Conclusions: As technology advances and digitalization in Romanian hospitals continues to develop, software developers and cybersecurity experts must establish a secure system for managing medical data, thereby enhancing public trust in telemedicine and facilitating patient adoption of new technologies, with the aim of preventing or detecting certain diseases in their initial stages. Targeted training programs, resource allocation, and international best practices are essential to reduce gaps in GDPR implementation, alignment with EU standards, and integration into the European Health Data Space (EHDS).

## Introduction

As healthcare becomes increasingly reliant on digital technologies, safeguarding patient data has emerged as a critical priority, particularly within the European Union (EU) under the General Data Protection Regulation (GDPR) [1].

In February 2024, a ransomware attack locked the databases of 20 hospitals in Romania, rendering the reporting of medical services and associated reimbursement to the state difficult for several days. Adhering to Article 32, letter (d) of the GDPR could have avoided this unfortunate incident [2].

Despite this incidence being an obvious infringement of GDPR, the National Authority for the Supervision of Personal Data Processing (ANSPDCP - dataprotection.ro) refrained from imposing any fines or corrective measures. In 2024, the National Authority did not conduct any oversight concerning GDPR compliance among hospitals in Romania, yet imposed three fines on private medical service providers for the unauthorized disclosure of personal data (e.g., diagnosis, treatment, test results, referrals, and hospitalizations), worth a total of 7,500 euros, following notifications from service providers in accordance with Article 33 of the GDPR.

In Romania, the incorporation of GDPR principles into the healthcare system seeks to bolster patient trust while simultaneously mitigating rising worries about data breaches and the abuse of personal information. The implementation of GDPR in Romania requires a comprehensive grasp of its provisions, especially in healthcare, which frequently involves the management of sensitive data.

Despite its importance, compliance with GDPR remains challenging in Romania’s healthcare sector. Smaller healthcare providers often lack the resources to achieve full compliance, while the rapid evolution of medical technologies compounds these difficulties by introducing new regulatory complexities.

The swift advancement of healthcare technologies hampers adherence, as medical personnel frequently struggle to balance innovation with strict regulatory constraints [3]. Thus, a thorough examination of this dynamic indicates a significant necessity for specialized education and training initiatives designed to improve GDPR understanding among healthcare stakeholders [4]. The success of GDPR implementation in Romania's medical industry ultimately depends on cultivating a culture of data protection and privacy awareness. This entails not only legal adherence but also the imperative for ongoing adjustment as digital environments transform. Implementing comprehensive training programs, coupled with continuous evaluation of compliance protocols, is crucial for reducing risks associated with data privacy violations.

Aligning GDPR principles with internationally recognized frameworks, such as the International Organization for Standardization (ISO) standards (e.g., ISO 27001:2022), can help establish a robust foundation for data protection, particularly in managing sensitive patient information.

This article aims to identify the key obstacles faced by Romanian healthcare leaders in implementing GDPR and proposes measures to ensure compliance with European directives, with a particular focus on the GDPR and the Network and Information Systems Directive (NIS 2). Partially addressing the NIS 2, this article underscores its complementary role in enhancing cybersecurity measures alongside GDPR compliance.

## Materials and methods

Participants were selected from a group of healthcare professionals attending the economics and healthcare management course. Inclusion criteria required participants to be actively employed in managerial roles within the Romanian public healthcare system, with representation from public hospitals, health ministries, and public health departments.

This study is based on healthcare professionals’ expertise analyzed through a privacy-focused survey using Google Forms (Google, Mountain View, CA) and presented in Appendices. The survey was distributed to 108 healthcare professionals enrolled in the economics and healthcare management course. A reminder email was sent to participants one week after the initial invitation to ensure a high response rate. After one week, the response rate was 49, and after an additional five days, the final response rate was 68%, with 74 completed surveys returned.

The survey was designed specifically for this cross-sectional study, incorporating both closed and open-ended questions. Expert validation was sought from professionals in healthcare management and GDPR compliance to confirm the relevance and appropriateness of the questions. Prior to distribution, the questionnaire was piloted with a small group (15 experts) of healthcare professionals to ensure clarity and comprehensibility. The experts' recommendations were related to form and presentation rather than content.

Adequate statistical tests were used to compare the responses across the demographic groups (e.g., gender and age), roles, and professions. All data were analyzed employing only descriptive statistics to summarize demographic data and assess trends in GDPR knowledge and implementation.

Informed consent was obtained from all participants prior to survey completion. Participants were assured that their responses would remain anonymous and confidential. No personally identifiable information was collected, with demographic data limited to age, gender, qualifications, and position within the healthcare system.

No prior approval was necessary from the Research Ethics Committee because the National Authority for Personal Data Processing has established exceptions for academic and research objectives from a legal standpoint. This complies with the stipulations of Article 12 lit. (a) 1 (human protection) of National Law 206/2004 (concerning ethical conduct in scientific research, technical development, and innovation) and Articles 7 and 8 of National Law 190/2018 (GDPR exceptions).

## Results

In Romania, the knowledge of GDPR among healthcare professionals remains an area marked by variability, reflecting a broader trend observed across the EU. Many practitioners demonstrate a foundational understanding of GDPR principles, primarily related to patient consent and data protection measures, yet gaps exist in practical applications, particularly in clinical settings. An exploratory survey regarding patients’ knowledge about data protection in the EU underscores that 80% identified correctly that the responsibility for the protection of personal data when the data are used for medical research is for the individual or entity that uses the data. This shows that EU citizens are aware of privacy concepts in light of the GDPR [5].

Demographics

The oldest participant in the survey was 66 years old, and the youngest was 22 years old, consisting of 55 females and 19 males, from a total of 108 targeted professionals. Among the 74 participants, there were 17 nurses, 32 doctors, and 25 individuals from other professions, including legal professionals, managers, and economists. Out of the 32 managerial roles, 15 were held by doctors, 14 by individuals from other professions, and two by nurses (Table 1).

**Table 1:** Participants’ frequency table by demographic groups.

Age (years)	Sex		Profession			Role		Age frequency
	Male	Female	Doctor	Nurse	Other profession	Management	Executive	
22	1	1	-	2	-	-	2	2
23	-	4	-	5	-	-	5	5
24	-	2	-	1	1	-	2	2
29	2	1	2	1	-	-	3	3
31	-	1	1	-	-	-	1	1
32	-	2	2	-	-	1	1	2
33	1	-	-	-	1	1	-	1
34	2	1	1	-	2	1	2	3
35	-	3	1	1	1	-	3	3
36	1	1	1	-	1	1	1	2
37	-	1	-	-	1	-	1	1
40	-	2	1	1	-	-	2	2
41	-	1	1	-	-	1	-	1
42	-	1	-	-	1	-	1	1
43	-	2	1	1	-	1	1	2
44	-	1	1	-	-	-	1	1
45	1	-	-	-	1	1	-	1
46	1	3	3	-	1	2	2	4
47	1	3	3	-	1	2	2	4
48	4	4	3	1	4	5	3	8
49	-	2	-	1	1	1	1	2
50	2	1	2	-	1	1	2	3
51	-	5	2	-	3	5	-	5
52	-	2	1	1	-	2	-	2
53	1	2	1	2	-	1	2	3
54	1	-	-	-	1	-	1	1
56	-	2	-	-	2	2	-	2
57	-	2	1	-	1	-	2	2
59	1	1	2	-	-	2	-	2
60	-	1	1	-	-	-	1	1
61	-	1	1	-	-	1	-	1
66	-	1	-	-	1	1	-	1
Frequency	19	55	32	17	25	32	42	-
Total	74		74			74		74

General knowledge of privacy and GDPR

**Figure 1:**
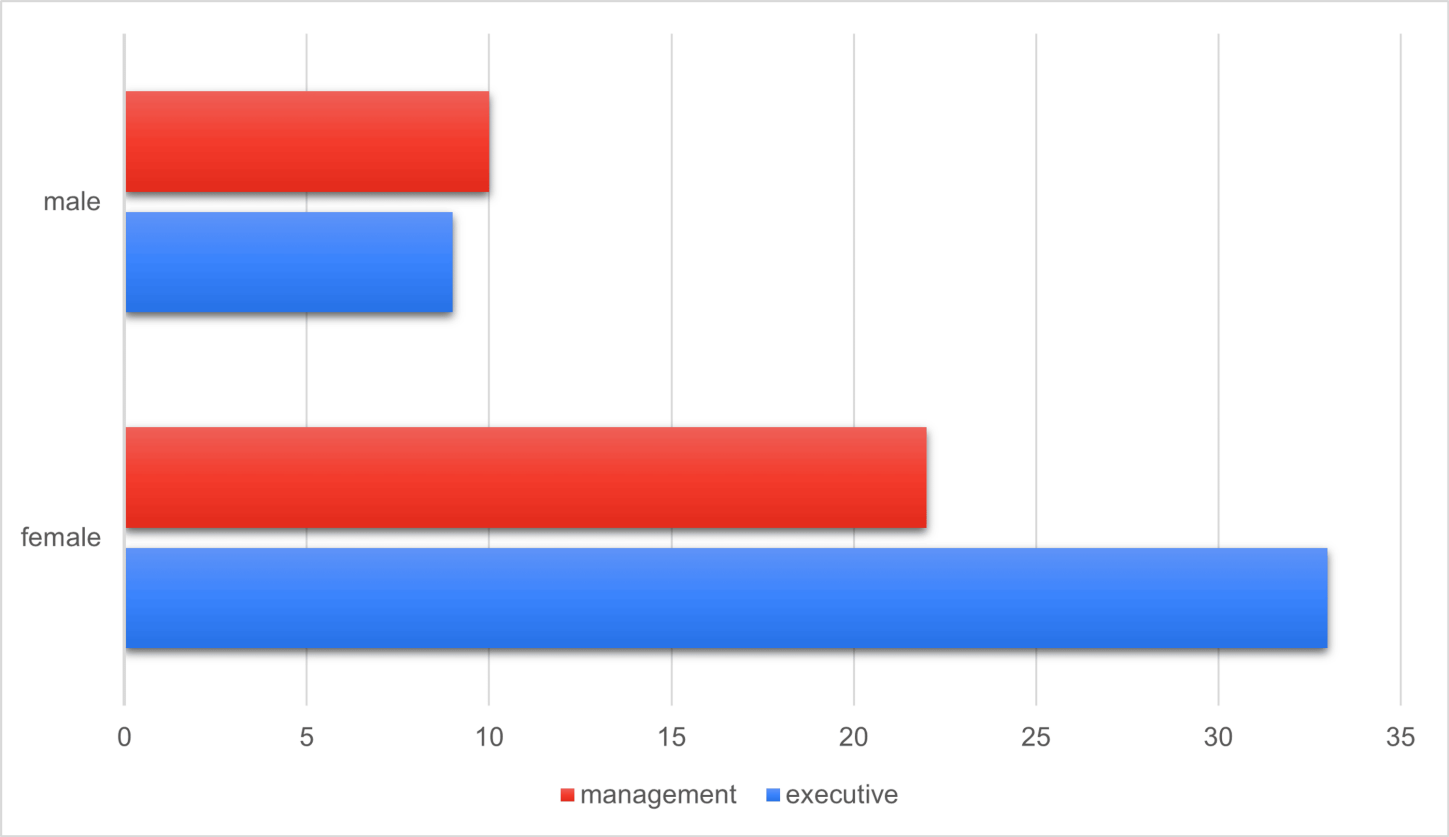
Gender of healthcare professionals.

Female healthcare professionals occupy management positions in their organizations more frequently than their male counterparts. Figure 1 illustrates the proportion of female professionals in management roles, highlighting their greater representation compared to male counterparts.

Despite healthcare workers' awareness of GDPR, about 65% of participants reported a thorough understanding of privacy after six years of its enforcement compared to Finland where the average for well-educated people is between 40% and 54% [6].

The findings indicate that while a majority of healthcare professionals report a foundational understanding of GDPR, practical gaps remain, especially in clinical settings. This discrepancy highlights the need for targeted educational initiatives and ongoing training to bridge the gap between theoretical knowledge and practical application. These results underscore the importance of tailored GDPR training programs to ensure that healthcare professionals can effectively implement data protection principles in their daily practices.

Participants included 74 professionals in clinical roles and 34 in administrative roles, with 52% occupying management positions underscoring that managers are in alignment with other EU countries regarding their understanding of privacy.

The next step was to assess the implementation of GDPR inside their organization, revealing that 81% of managerial roles indicated compliance with GDPR.

The next phase was evaluating the challenges of implementing GDPR within their organization, which concluded that only 22% of managerial positions indicated that compliance with GDPR was really challenging as evidenced by their responses on a scale from 1 (very easy) to 10 (extremely difficult).

In-depth knowledge of privacy and GDPR

The objective of the second segment of the survey was to evaluate the comprehensive understanding of the fundamental principles of GDPR and privacy as articulated in national and international legislation. Healthcare practitioners were queried regarding the essential aspect of patient privacy, specifically, informed consent for medical treatment as stipulated in Articles 13-20 of Romanian Law 46/2003, and consent for the processing of medical data, as outlined in Article 6 of the GDPR. This study shows that 34% of interviewees perceived no distinction between consent under national and international legislation or were unable to provide a response.

The most critical aspect of informed consent is its mandatory nature, and GDPR consent is required for other purposes (secondary utilization) of health data processing. Article 9 serves as the foundation for healthcare providers' processing of health data under GDPR, and only information specified in Article 13 is required.

This indicates that 19% of managerial positions lack comprehension of the essential distinctions between national and international laws in terms of privacy for medical records or sensitive health data.

When inquired whether the disclosure of a patient's medical information to other interested parties (such as family members or the media) defines a medical error (a breach of confidentiality) that could be perceived as malpractice, 69% of respondents indicated that they regard it as a medical error and over 50% of those were holding management positions and had ages between 32 and 61 years old.

The study did not reveal any significant disparities between elder and younger managers in their comprehension of the distinctions between data confidentiality concepts.

During data sorting, an intriguing observation emerged: a specific management position, although being a jurist, failed to recognize the disclosure of medical information as a violation of private data regulations.

The next phase entailed evaluating their abilities to examine the effects of digitalization in public hospitals on healthcare privacy. We found that 82% of the participants believe that digitalization reduces the risk of privacy from a GDPR perspective, given that 43% of the total participants have management positions.

It is also possible to observe that 18% of managers do not know or believe that digitization has no influence on the privacy of patient data whatsoever, while 16% of managers believe that risks increase in tandem with digitalization.

Despite the growing integration of digital technologies in healthcare, the results indicate a disconnect between perceived and actual risks to data privacy. While digitalization has the potential to enhance data protection through secure systems, many healthcare professionals remain unaware of its impact on patient privacy. This suggests the need for better communication and training to help healthcare professionals understand the relationship between digitalization and GDPR compliance, and to mitigate potential risks associated with emerging technologies such as AI.

The final two questions are open-ended and focus on exceptions and the challenges healthcare professionals face in their daily duties.

A total of 53% of healthcare professionals consider that exceptions to privacy under GDPR may be permitted for public health concerns. This suggests that 47% of healthcare professionals in Romania possess a fundamental deficiency in understanding privacy concepts and the conditions under which exceptions may be allowed, with ethical concerns cited as a significant rationale for not permitting exceptions.

The most intriguing question in the study was to identify the most substantial barrier or obstacle that GDPR has posed in their daily duties. Older professionals showed greater reluctance in adopting technology due to issues such as managing multiple credentials and insufficient IT support for database maintenance and cybersecurity responses.

The challenges recognized by healthcare professionals in the implementation of GDPR requirements in their organizations are described in Table 2.

**Table 2:** Challenges recognized by participants categorized by topic. IT&C: information technology and communication; GDPR: General Data Protection Regulation.

Administrative burdens	Technological barriers	Communication issues
Accountability	Databases blocking (IT&C failures)	Communication – sending files using social networks and other communication apps
High volume of consent on print papers	Stress when sharing information because of the lack of control after sending sensitive information	Less time spent with patients because of administrative tasks
Time consuming for consent forms	Phone information about patients and oral consent	Pseudonymize personal data (first and last name) confuses healthcare patients and medical staff
Publishing students' grades after exams	Restrict and limit access to specific health data for emergency situations	Abusive use of GDPR for not reporting different information to public healthcare departments
Lack of notification in case of data breaches	Usage of user and different passwords for all apps in hospitals	Institutional collaboration and collaboration because of the lack of training regarding GDPR
	Sending dozens of copies containing medical information	

To address the administrative challenges identified in the study, particularly the time-consuming nature of paper-based consents and GDPR forms, healthcare organizations should prioritize the adoption of digital document management systems. These systems could streamline the process, allowing for secure, efficient data collection and storage, and reducing the burden on healthcare professionals. Such a move would not only support GDPR compliance but also improve patient care by freeing up time for more direct interactions between healthcare providers and patients.

Ethical challenges, such as the pseudonymization of patient data and the reluctance to share information with family members due to GDPR restrictions, have emerged as significant barriers in healthcare. These concerns reflect a need for clearer guidelines on the interpretation of GDPR in sensitive situations and for policies that balance data privacy with the practical needs of healthcare professionals. The implementation of GDPR should not hinder communication and patient care but should instead ensure that privacy protections are integrated into daily practices without impeding the provision of medical services.

Through meticulous examination of the comments concerning the difficulties in implementing GDPR, we concluded that 34% of the participants see education and training as a critical issue requiring attention. Moreover, 50% of all management positions agree that this issue may be addressed by training administrative personnel under the guidance of an expert, as the function of the data protection officer (DPO) is formally assigned, with GDPR responsibilities typically delegated to an individual who has often completed a basic knowledge course in the area. Furthermore, this individual performs various responsibilities besides the fundamental job description.

In Germany, a study on consent for data processing for research purposes was conducted by querying GDPR experts, IT professionals, and patients. The study identified a number of challenges that are similar to those identified in this study. In hospitals, patients feel pressure to provide consent for research as a prerequisite for routine investigations, and they are concerned about the staff's lack of education in data security. IT specialists identified the withdrawal of consent as a technical challenge, as well as data security in the context of transfers to non-EU countries [7].

A study on healthcare collaboration examined the legal requirements and challenges associated with implementing the European Health Data Space (EHDS) from a GDPR perspective, identifying several issues related to the responsibility of data sharing in cloud deployment. From the standpoint of pseudonymization and interoperability, the GDPR permits national authorities to implement derogations inside EU member states, potentially leading to ambiguity over accountability in the event of security breaches. Simultaneously, there exists concern that individuals may be unaware of the reasons for which their medical data have been accessed, thereby fostering distrust in the healthcare system over the administration of their medical information [8].

In Romania, reducing bureaucracy by digitizing internal flows and enhancing patient trust in the medical system by improving communication with the patient by dedicating time to clarifying the results and treatment steps to be followed will be achieved by addressing these challenges for GDPR compliance. Simultaneously, the heightened trust of patients will result in more rapid adoption of smart devices for the purpose of monitoring specific parameters through telemedicine services, thereby preventing unnecessary congestion in the emergency room.

## Discussion

GDPR legal requirements for health data processing in Romania

While the GDPR prioritizes data protection, it recognizes the importance of processing data for public health purposes, allowing for the necessary use of data while still ensuring adequate safeguards for individuals' privacy. The international regulation provides several legal bases for processing health data without an individual's explicit consent, specifically for public health reasons.

Article 6(1)(e)

Processing is necessary for the performance of a task carried out in the public interest or in the exercise of official authority vested in the controller. This might cover tasks related to public health monitoring and surveillance, health inspections, and management of healthcare services by public authorities.

Article 9(2)(i)

Processing is necessary for reasons of public interest in the area of public health, such as protecting against serious cross-border threats to health. This often relates to activities like preventing or controlling the spread of diseases, managing healthcare systems, ensuring the quality and safety of healthcare, and conducting public health research.

Given that the Romanian healthcare sector is thoroughly regulated, healthcare practitioners cannot invoke malpractice law, as all matters are now covered by Article 653 of National Law 95/2006, Article 37(1) of National Law 46/2003, and ethical guidelines. This renders them susceptible to legal misinterpretation, and frequently they decline to discuss medical results and therapy with family members or relatives, citing GDPR constraints. One of the most exasperating GDPR measures [9] from the perspective of healthcare professionals, implemented extensively in Romanian hospitals, is the pseudonymization [10] of personal data (first and last name become identity (ID)) by generating barcodes or ID numbers, which renders patients feeling less human and more like objects or inventory assets. Despite the technical adherence to confidentiality, patients want another compensatory measure. Patient associations for various ailments are consulted during the legislative process, irrespective of the nature of the proposed modifications by the initiators; however, no such actions have been undertaken thus far.

While digitalization is a hot topic in Romania, patients admitted to hospitals are confronted with an extensive array of printed documents, predominantly comprising consents for various medical operations and the processing of health data. None of these documents guarantee that the patient's medical data are adequately protected from unwanted access or disclosure and preserve the confidentiality of the doctor-patient relationship.

Studies [7,8] indicate that patients' primary concern is data security, which is exacerbated by insufficient education and expertise in cybersecurity, as pointed out by numerous media reports on cyberattacks targeting hospital infrastructure. Significant investments in IT and communication infrastructure, staff training, and the establishment of collaboration partnerships with public entities, such as universities, and private sector partners, including IT and cybersecurity firms, may enhance patients' perceptions of hospitals' data protection capabilities.

Additionally, there are few regulations or concerns regarding genuine GDPR [11] compliance in Romania. The vast majority of medical institutions rely on fragile compliance based on consent forms that frequently prove useless and privacy policies that are copied from the internet and have no relevance to the institution in question.

In accordance with Article 20 of the GDPR (portability), all systems that control patient data related to diagnoses, treatments, and analyses must be capable of automatically exporting structured data that can be transmitted through the patient's electronic health record or the EHDS.

Gender and age disparities

In accordance with Directive (EU) 2022/2381, which aims to enhance gender balance among directors of listed companies and implement relevant measures, it is evident from a gender point of view that the proportion of females in management roles is double that of males.

This study clearly indicates that women more frequently hold managerial positions than men in the healthcare sector, despite the results of the National Agency of Public Servants’ (NAPS) study conducted in 2023, concluding that men are promoted to management roles more frequently than women. The NAPS study indicates a modest increase in the proportion of women in management positions over the past five years (2019-2023), from 19.5% to 33.9%, and also identifies several advantages of women in management positions, including the ability to create a positive image of the organization, promote diversity and inclusion, develop empathy toward employees, and make drastic decisions more quickly in times of crisis [12]. The abilities of women in management positions can be significant in enhancing working conditions, raising the attractiveness of employment within the organization, and increasing people's trust in public institutions and institutional transparency.

Age disparities among participants revealed differing perceptions of digitalization risks. Older professionals viewed digitalization as low-risk, whereas younger professionals emphasized risks associated with interconnected systems and public networks [13-15]. Elder doctors are particularly concerned about the excessive use of usernames and passwords to access patient data, the ability to retrieve specific health information during emergencies, and the potential for IT failures.

Studies regarding the digitization paradigm in Romanian hospitals are currently being conducted to advance this field and conduct additional research like WP3 human factors in healthcare cyber security from the Romanian Cyber Care Health (RO-CCH) European project ending on 31st of December 2024, coordinated by the Romanian National Cyber Security Directorate (DNSC) [16].

As emphasized in numerous studies, healthcare professionals are at the forefront of managing patient data and face substantial legal obligations to safeguard this information from security risks [16-18]. On the other hand, patients’ perspective regarding the use of technology in healthcare is influenced by age, digital skills, travel distance, education level, and severity of the disease [19-21].

From the GDPR and NIS 2 perspective, a holistic strategy that includes physical, technical, and administrative controls, along with ongoing education and awareness programs, is essential for equipping personnel to identify and mitigate cyber threats, thereby protecting medical data and maintaining the integrity of healthcare systems [22].

Challenges and opportunities

The primary idea is that only 74 out of 108 respondents completed the survey, indicating that over 30% of healthcare professionals are not inclined to comply with the GDPR.

This also suggests that greater awareness is necessary to underscore the significance of privacy when health data are gathered, processed, shared, revealed, or transferred to patients or household members via the internet through various applications [23]. The Romanian government should implement or create a telemedicine application to enhance communication between physicians and patients. This application must be able to transmit medical analyses (PDF files or structured health data) and provide audio-visual communication to ensure a clear comprehension of the physician's message.

The integration of advanced technologies, such as AI for diagnostic purposes, introduces further complexity, underscoring the necessity for comprehensive training and effective policy frameworks that emphasize both GDPR compliance and ethical standards in data handling. As evident in studies incorporating explainable AI in medical imaging, success hinges on an informed workforce equipped with the tools to navigate privacy regulations effectively [24]. These dynamics highlight the need for enhanced educational initiatives targeting the subtleties of GDPR, to empower professionals in maintaining patient trust while fostering innovation in the healthcare sector.

Additionally, physicians are also burdened by the completion of an extensive amount of documentation, which consumes time that they would prefer to allocate to elucidating the diagnosis and treatment to the patient. It is recommended that all healthcare providers implement a digital document management system that digitizes processes from the patient's admission to their discharge. This will enable the patient to complete the admission or discharge process with a digital signature and a few keystrokes.

The implementation of the GDPR in Romanian healthcare presents both challenges and opportunities. Challenges may include resource constraints (the need for significant investments in technology, infrastructure, and human resources), cultural barriers (overcoming resistance to change and promoting a data protection-centric culture), and complexity of healthcare data (the diverse and sensitive nature of healthcare data that can make compliance challenging).

Opportunities that come with GDPR compliance may include improved patient trust (enhancing public confidence in the healthcare system through robust data protection measures), innovation (stimulating the development of new technologies and practices that support data privacy and protection), international collaboration (fostering cooperation with other EU member states and international partners on data protection issues), and GDPR awareness for secondary usage of personal data in public health sector.

Proposed solutions for GDPR compliance in Romanian hospitals

To address the obstacles presented by digitalization and GDPR compliance requirements (Recitals no. 78, Art. 25 & 32 from the GDPR) [25], hospital managers in Romania should implement a series of urgent measures. Implementation should occur in phases, guided by a strategic plan of action, prioritizing steps that substantially mitigate data protection risks, as shown in Table 3 and detailed in Appendices.

**Table 3:** Prioritized solutions for GDPR compliance. GDPR: General Data Protection Regulation; ISO: International Organization for Standardization; EHDS: European Health Data Space.

Priority	Organizational measures	Technical measures
1	Appoint or contract privacy professionals (cybersecurity and data protection)	Update all operating systems and software used in daily routines
2	Establish partnerships with public and private entities	Upgrade IT infrastructure
3	Recruit more technical personnel	Training personnel with the most recent technologies in cybersecurity and infrastructure administration
4	Contract and adherence to ISO certifications (e.g., 9001 and 27001)	Continuous oversight of the IT infrastructure along with the execution of procedures for ISO standards compliance
5	Develop educational programs for patients regarding the use of smart devices for the benefit of active monitoring of health parameters	Conducting simulations for IT infrastructure resilience and biannual cybersecurity evaluations
6	Establish protocols for EHDS interconnection or AI integrations	Prepare IT infrastructure for cloud interconnection/migration or AI integration

This study supports previous research from EU member states, indicating that the primary concern of individuals, data security, comes from a deficiency in cybersecurity skills. An immediate remedy would be to train the personnel in data protection; nevertheless, the circumstances of hospitals in Romania are unique due to inadequate expenditures in IT infrastructure over the past decade.

A thorough review of security breaches in Europe from 2012 to 2022 also revealed a range of issues, similar to those in the current study. Human mistakes stemming from insufficient training, outdated operating systems that remain without security patches, lack of investment owing to governmental underfunding, and technical advancements are among the issues recognized at the European level that are equally evident in the Romanian medical system [26].

To increase the patients’ trust in the healthcare system, the establishment of a secure environment within Romanian hospitals should be a top priority, as the information technology and communication (IT&C) infrastructure is outdated and the technical staff is poorly compensated and lacks cybersecurity expertise.

The subsequent actions involve forming partnerships with universities and private enterprises in cybersecurity to initiate European projects aimed at enhancing IT infrastructure and recruiting and training technical staff in cybersecurity to guarantee the cyber resilience of hospital IT systems against malicious attacks.

Adherence to ISO/International Electrotechnical Commission (IEC) 27001:2022 and ISO/IEC 27018:2019 standards proves that organizations have in place security measures and procedures to protect data and enhance patient trust in the healthcare system, facilitating the use of technology, thereby smoothing the transition to telemedicine.

With the integration of smart devices for monitoring daily metrics, government-supported hospitals can initiate campaigns for the promotion of their safe and proper usage. Consequently, public health initiatives or policies can be formulated for the prevention of many diseases that can be identified in their early stages by technological assistance. Establishing a legislative framework enables the government to deploy telemedicine platforms that automatically incorporate data from smart devices, facilitating AI-assisted general suggestions for patients and alerts for physicians to ensure accurate diagnosis and personalized treatments [27]. Additionally, these data may be transmitted or integrated into EHDS or processed for secondary research purposes, such as in the pharmaceutical industry.

Study's strengths and limitations

One of the key strengths of this study is its focus on healthcare professionals' experiences and challenges with GDPR implementation in Romania. By combining both quantitative and qualitative data, the study provides a comprehensive understanding of the barriers and opportunities associated with GDPR compliance in the healthcare sector. Additionally, the study’s findings offer valuable insights into the intersection of data privacy and healthcare technology, which can inform future policy and training initiatives.

A limitation of this study is the relatively small sample size (108 participants), which may not fully represent the broader healthcare sector in Romania. The fact that the participants were all enrolled in a specific healthcare management course (mandatory for management roles in the public healthcare sector) could introduce selection bias, as the sample may be skewed toward professionals with more interest or experience in healthcare administration. Additionally, as a self-report survey, the study is susceptible to response bias, where participants may have overestimated their understanding of privacy or may have felt compelled to provide socially desirable answers. These factors limit the generalizability of the findings to all healthcare professionals in Romania.

Future research could explore the experiences of non-managerial healthcare professionals, as well as the impact of GDPR requirements on patient care and overall healthcare outcomes. Additionally, comparative studies between different EU member states could provide insights into the factors influencing GDPR compliance in diverse healthcare settings.

In future studies, it would be beneficial to include a larger and more diverse sample of healthcare professionals, particularly those in non-managerial roles, to gain a more comprehensive understanding of GDPR implementation across the healthcare sector. Additionally, employing a mixed-methods approach that combines surveys with in-depth interviews could provide richer, more nuanced insights into the challenges and opportunities faced by healthcare professionals in complying with privacy regulations.

## Conclusions

As new technology arises, a collective effort involving policymakers, healthcare providers, patients, and privacy specialists is crucial to ensure GDPR compliance. The study findings reveal that while healthcare professionals generally understand GDPR principles, they face significant challenges in applying these principles within their organizations. The data underscore the need for specialized training programs, enhanced resources, and clearer procedural guidelines to support effective GDPR implementation. These results directly inform our conclusion that a more robust approach to education and organizational change is critical for improving GDPR compliance in Romania's healthcare sector.

The significant barriers identified by participants, such as the overwhelming administrative burden of consent forms and concerns over data security, suggest that GDPR compliance is straining healthcare professionals' ability to provide optimal patient care. These challenges highlight the need for streamlined processes and enhanced cybersecurity measures to ensure both compliance and the effective delivery of healthcare services. It is important to note that the sample in this study primarily consisted of healthcare professionals enrolled in a specific management course, which may limit the generalizability of the findings to the wider healthcare workforce in Romania. Therefore, while the conclusions drawn are insightful, they should be interpreted with caution until further research is conducted involving a broader and more representative sample. As the healthcare sector continues to digitize, the study findings indicate a growing concern among healthcare professionals about data breaches and cybersecurity. The conclusion emphasizes the urgent need for advanced technological solutions, such as secure data management systems and better-trained IT personnel, to mitigate these risks and align with GDPR compliance. These technological advancements should be prioritized to safeguard patient data while fostering innovation in healthcare delivery.

## Appendices

Survey

**Table 4:** Survey on the awareness of personal data privacy regulations among public health professionals.

Section 1 – Personal data	
1. Age	User input
2. Gender	Male/female
3. Medical specialty	User input
4. Role you currently occupy	Management/executive
5. Geographical area of your current job	User input
Section 2 - General knowledge of privacy and GDPR	
Scale 1 (not at all) to 10 (very well)	
6. To what extent do you believe you are familiar with the General Data Protection Regulation (GDPR)?	Scale 1 to 10
7. What is your assessment of the implementation of GDPR within your organization?	Scale 1 to 10
8. What is your assessment of the challenges associated with adopting GDPR within your organization?	Scale 1 to 10
Section 3 - In-depth knowledge of privacy and GDPR	
9. Do you believe there are distinctions between informed patient consent and GDPR?	Yes/No/Don’t know
10. Do you consider that the disclosure of a patient's medical data to third parties (such as family members or the press) constitutes a breach of confidentiality that can be classified as malpractice?	Yes/No/Don’t know
11. Considering your personal and professional experience, what do you perceive as the influence of hospital digitization on the privacy of medical data?	Low risk/high risk/no impact/don’t know
12. Do you consider that, in the interest of public health, exceptions can be made to the confidentiality of individuals' medical data for the common good?	User input
13. What is the most significant challenge or obstacle that the GDPR has created for your daily operations?	User input

Details of proposed solutions for GDPR compliance

Acquiring processing and storage equipment (many hospitals still utilize Windows 8 workstations and numerous servers that are over five years old in operation).

Acquiring specialized equipment and cybersecurity solutions to protect medical data in hospitals (many hospitals own equipment with firewall functionalities, although they are often inadequately set).

Regularly executing security upgrades for all operating systems and devices utilized in hospitals (certain workstations and servers remain purposely unpatched due to specific applications utilized for controlling particular operations that are not compatible with the current system or library packages).

Upgrading the network infrastructure in hospitals (certain hospitals still utilize cat5 cables instead of fiber optics cable and backbone core switches and routers).

Securing a role with duties in cybersecurity as a chief information security officer (CISO) to join the hospital's board of directors. Currently, a network and information security (NIS) officer is appointed at the hospital level, a cumulative role assigned to an individual from the IT department, who is frequently untrained, unpaid, and lacks the requisite authority to enforce a range of security measures for the protection of sensitive data.

Recruiting more technical personnel in the IT&C departments of hospitals, which are inadequately staffed in the majority of hospitals in Romania.

Providing financial remuneration for the retention of IT&C specialists by engaging them in national and international research initiatives.

Training and preparing IT&C professionals for the utilization of acquired systems and equipment to maximize their potential capabilities.

Performing tests or simulations to evaluate the responsiveness to a security incident or disaster recovery scenario.

Prepare educational programs for digital health (eHealth) aimed primarily at enhancing digital competencies for doctors.

Facilitating demonstration sessions for patients in hospitals regarding the utilization of diverse medical gadgets or software applications to enhance technological adoption for preventive measures.

Performing biannual security evaluations (by DNSC (Directoratul Național de Securitate Cibernetică) or accredited security auditors) to ascertain the condition of the hospitals' IT and communications infrastructure.

Accrediting diverse hospital systems and processes in accordance with international ISO standards to enhance public trust in the capacity to safeguard medical data.

Cooperation with the National Supervisory Authority for Personal Data Processing to formulate a legislative initiative for the secondary usage of medical data in Romania.

Establishing partnerships with universities and private companies possessing technological or medical expertise for improving and incorporating cutting-edge technologies, including artificial intelligence and blockchain, in both the hardware and software applications used within hospitals.

## References

[1] Jeyaraman N, Ramasubramanian S, Yadav S, Balaji S, Muthu S, Jeyaraman M (2024). Regulatory challenges and frameworks for fog computing in healthcare. Cureus.

[2] (2024). DNSC. Ransomware attack against Romanian hospitals. https://dnsc.ro/citeste/atac-cibernetic-ransomware-spitale-Romania.

[3] Cambronero ME, Martínez MA, Llana L, Rodríguez RJ, Russo A (2024). Towards a GDPR-compliant cloud architecture with data privacy controlled through sticky policies. PeerJ Comput Sci.

[4] Williamson SM, Prybutok V (2024). Balancing privacy and progress: a review of privacy challenges, systemic oversight, and patient perceptions in AI-driven healthcare. Appl Sci.

[5] Lalova-Spinks T, Saesen R, Silva M, Geissler J, Shakhnenko I, Camaradou JC, Huys I (2024). Patients’ knowledge, preferences, and perspectives about data protection and data control: an exploratory survey. Front Pharmacol.

[6] Kyytsönen M, Vehko T, Jylhä V, Kinnunen UM (2024). Privacy concerns among the users of a national patient portal: a cross-sectional population survey study. Int J Med Inform.

[7] Wiertz S, Boldt J (2024). Ethical, legal, and practical concerns surrounding the implemention of new forms of consent for health data research: qualitative interview study. J Med Internet Res.

[8] Kertesz F (2024). Collaboration in healthcare: implications of data sharing for secondary use in the European Union. Eur J Health Law.

[9] (2024). Ministry of Education. For the first time since the entry into force of the GDPR, the names and surnames of candidates for national exams will be published in anonymized format. (Article in Romanian). https://www.edu.ro/pentru-prima-dat%C4%83-de-la-intrarea-%C3%AEn-vigoare-rgpd-numele-%C8%99i-prenumele-candida%C8%9Bilor-la-examenele.

[10] (2025). European Data Protection Board. Guidelines 01/2025 on pseudonymisation. https://www.edpb.europa.eu/system/files/2025-01/edpb_guidelines_202501_pseudonymisation_en.pdf.

[11] (2024). The National Supervisory Authority For Personal Data Processing. Primary legislation. https://www.dataprotection.ro/index.jsp?page=legislatie_primara&lang=en.

[12] (2024). National report of management roles in the public sector. (Article in Romanian). https://www.anfp.gov.ro/R/Doc/2024/Rapoarte/Raport%20privind%20managementul%20func%C8%9Biei%20%C8%99i%20func%C8%9Bionarilor%20publici%202023.pdf.

[13] Jerry-Egemba N (2024). Safe and sound: strengthening cybersecurity in healthcare through robust staff educational programs. Healthc Manage Forum.

[14] Ness S, Khinvasara T (2024). Emerging threats in cyberspace: implications for national security policy and healthcare sector. J Eng Res Rep.

[15] Al-Qarni EA (2023). Cybersecurity in healthcare: a review of recent attacks and mitigation strategies. Int J Adv Comput Sci Appl.

[16] (2024). Romanian Cyber Care Health - RO-CCH. https://dnsc.ro/pages/proiect-ro-cch.

[17] Elendu C, Omeludike EK, Oloyede PO, Obidigbo BT, Omeludike JC (2024). Legal implications for clinicians in cybersecurity incidents: a review. Medicine (Baltimore).

[18] Hoffmann S, Ott M, Bobbert F, Jacoby J, Henes M (2024). Data protection concerns are a statistically significant reason for refusal: exploratory study of the patient perspective on telemedicine services based on 735 gynecological surgery patients. Digit Health.

[19] Alhammad A, Yusof MM, Jambari DI (2025). Evaluating applied security controls for safeguarding medical device-integrated electronic medical records. J Eval Clin Pract.

[20] Mohammad Amini M, Jesus M, Fanaei Sheikholeslami D, Alves P, Hassanzadeh Benam A, Hariri F (2023). Artificial intelligence ethics and challenges in healthcare applications: a comprehensive review in the context of the European GDPR mandate. Mach Learn Knowl Extr.

[21] Jin MX, Kim SY, Miller LJ, Behari G, Correa R (2020). Telemedicine: current impact on the future. Cureus.

[22] Arabsorkhi A, Khazaei E (2024). Blockchain technology and GDPR compliance: a comprehensive applicability model. Int J Web Res.

[23] Carmi L, Zohar M, Riva GM (2023). The European General Data Protection Regulation (GDPR) in mHealth: theoretical and practical aspects for practitioners' use. Med Sci Law.

[24] Zaguir NA, de Magalhães GH, de Mesquita Spinola M (2024). Challenges and enablers for GDPR compliance: systematic literature review and future research directions. IEEE Access.

[25] (2024). Regulation (EU) 2016/679 of the European Parliament and of the Council. https://eur-lex.europa.eu/legal-content/EN/TXT/HTML/?uri=CELEX:32016R0679.

[26] Ewoh P, Vartiainen T (2024). Vulnerability to cyberattacks and sociotechnical solutions for health care systems: systematic review. J Med Internet Res.

[27] (2024). European Data Protection Board. Opinion 28/2024 on certain data protection aspects related to the processing of personal data in the context of AI models. https://www.edpb.europa.eu/system/files/2024-12/edpb_opinion_202428_ai-models_en.pdf.

